# Integration of Mobile Devices and Point-Of-Care Diagnostic Devices—The Case of C-Reactive Protein Diagnosis

**DOI:** 10.3390/diagnostics9040181

**Published:** 2019-11-08

**Authors:** Xin-Fang Wu, Ching-Fen Shen, Chao-Min Cheng

**Affiliations:** 1 Institute of Biomedical Engineering, National Tsing Hua University, Hsinchu 300, Taiwan; xinfangwu.tina@gmail.com; 2 Department of Pediatrics, National Cheng Kung University Hospital, College of Medicine, National Cheng Kung University, Tainan 704, Taiwan

In recent years, the misuse and overuse of antibiotics has promoted antibiotic resistance, which has now become a global public health concern. Among all prescribers, family practitioners are responsible for providing the majority of antibiotics prescriptions. Patterns of misuse are all related to each other and include not completing the full dosage of antibiotics provided, storing antibiotics for future use, sharing antibiotics, and taking antibiotics without a prescription. Not taking the antibiotics as prescribed not only wastes valuable medical resources but also exacerbates the spread of antibiotic resistance by strengthening bacterial strains. Appropriate antibiotic prescription practices and uses are essential for reducing the growing trend toward antibiotic resistance. 

Recent research conducted in the United Kingdom by Christopher C. Butler, et al. [1] from January 2015 to February 2017, showed that point-of-care (POC) testing of C-reactive protein (CRP) used to assess acute exacerbations of chronic obstructive pulmonary disease (COPD) in primary care can safely reduce the use of antibiotics. This trial involved 653 patients from 86 general medical practices who were 40 years of age or older, had a diagnosis of COPD in their primary care clinical record, and were presented with an acute exacerbation of COPD with at least one of the Anthonisen criteria, including increased dyspnea, increased sputum volume, and the presence of purulent sputum. Before being randomly assigned into two groups, these patients were asked to complete a self-administered Clinical COPD Questionnaire to provide an assessment of COPD-related health, and the European Quality of Life-5 Dimensions-5 Level Questionnaire (EQ-5D-5L), measuring the adverse effects of antibiotics, health care utilization, and health utility. Randomization was used to divide the patients at a 1:1 ratio into one of two groups: (1) a CRP-guided group; and, (2) a usual-care group. Clinicians performed a CRP POC test as part of the CRP-guided group patient assessment and prescribed antibiotics based on the official guidance and their interpretation of CRP test results. The usual-care group did complete a CRP POC test. All patients underwent follow-up via telephone calls and face-to-face consultations. After 6 months, patients were asked to complete a Chronic Respiratory Disease Questionnaire (CRQ-SAS) and an EQ-5D-EL to determine disease-specific, health-related quality of health. Results indicate that fewer patients in the CRP-guided group reported antibiotic use, and fewer received antibiotic prescriptions at their initial consultation. In terms of general health status, the CRP-guided group had a higher health state score than the usual-care group, and the adjusted mean CRQ-SAS test difference between groups, which was small, indicated that there was no worsening of COPD-related health status. These pieces of evidence suggest that including a CRP POC test as part of the assessment for exacerbation of COPD in primary care may reduce patient-reported use of antibiotics as well as the prescribing of antibiotics by clinicians.

We believe that this research highlights the importance for providing more efficient medically based information to assist clinicians in gaining insight into exact patient health conditions. We recognize that whole blood tests in hospital settings are relatively costly and time-consuming, however, economical POC testing options exist that might facilitate more efficient initial examinations and more productive ultimate outcomes [2,3]. Our research group, for example, has focused on the development of an economical, paper-based CRP test that can be used in combination with a smartphone-based application to provide rapid and easily assessed CRP levels from whole blood (as shown in Figure 1). 

Our study has a two-fold aim. We have already developed a new format for producing paper-based CRP detection devices based on the length difference between the control and test paper-based channels (versus the conventional immunoassay approach). This new CRP detection device uses an inflammation-based detection approach. It leverages a latex agglutination reaction in response to C-reactive proteins to produce a quantifiable response (stain length) that can be observed with the naked eye. By comparing the difference in stain lengths between the two channels of our device, we can determine the level of C-reactive proteins in whole blood. This paper-based CRP detection device is fabricated via a wax printing method that defines hydrophobic boundaries within a filter paper substrate. Our process reduces manufacturing and assay costs, speeds up operation, and can be used to purify and chromatographically correct the interference caused by whole-blood components using only a tiny sample of whole blood (only 5 μL) by relying on the hydrophilicity of filter paper [4]. Secondly, we have developed a smartphone-based application that allows users to both record and analyze the length differences between channels of our device, calculate the CRP level, compare results with the official guidelines, and then display the final diagnostic results to the end-user. By taking CRP diagnosis capacity away from clinical setting requirements and putting the first analytical step into the hands of home users and first-responders, patients and family practitioners are provided a better understanding of conditions and a better road map for improved health.

In an era in which smartphone use is ubiquitous, using mobile devices for disease diagnosis, prevention, and management is a promising and foreseeable future. Integration between mobile devices and POC tools could empower patients and provide rapid and accurate decision-making evidence for efficient diagnosis and subsequent care, especially in regards to the misuse or overuse of antibiotics. We believe that the study on the integration of the point-of-care diagnostic devices with a smartphone application (mobile devices) can be applied to facilitate a wide range of potential applications in POC diagnostics and handheld detection device development.

## Figures and Tables

**Figure 1:**
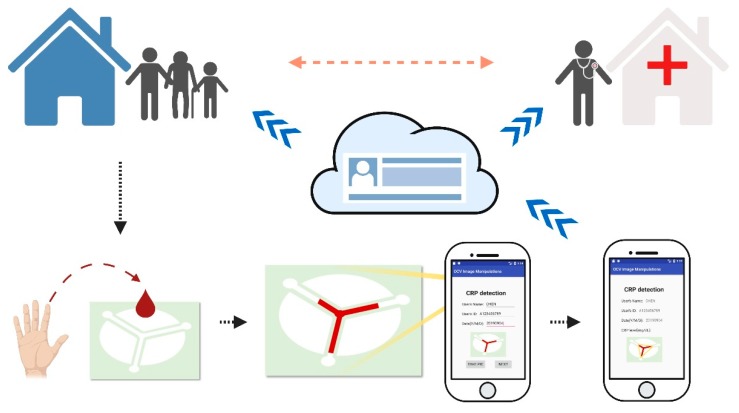
Schematic of the integration of point-of-care diagnostic tools and mobile devices. This figure presents the conceptual view of the clinical data flow of the point-of-care diagnostic tools and mobile devices in case of the C-reactive protein diagnosis that we have approached. Once the users place a drop of whole blood onto the diagnostic device, the whole blood would flow through the three channels. The users then could use the mobile application (APP) we have developed to take a picture of the diagnostic device with the final diagnostic result. The mobile application could help users both record and analyze the length difference of the diagnostic device, automatically compare with the guideline, tell the users the result and store the result in the cloud storage through the internet. In the cloud, the diagnostic result could be shared with the clinicians, enabling the clinicians to immediately gain insights about the patient situations without the patients needing to be near or even in the hospital.
